# Quantification of the Light Subunit of Neurofilament Protein in Cerebrospinal Fluid of Huntington’s Disease Patients

**DOI:** 10.1371/currents.hd.280c8f9f7d9fa4f7f0c883d9f8e807da

**Published:** 2018-08-31

**Authors:** Natalia Szejko, Carmen Picón, Juan García-Caldentey, Justo Garcia de Yebenes, Jose Carlos Alvarez-Cermeño, Luisa Maria Villar, José Luis López-Sendón Moreno

**Affiliations:** Department of Neurology, Medical University of Warsaw, Poland; Department of Bioethics, Medical University of Warsaw, Poland; Servicio de Inmunología. Hospital Ramón y Cajal. Madrid. Spain; Servicio de Neurología. Hospital Ramón y Cajal de Madrid. Spain; Servicio de Neurología. Hospital Ramón y Cajal de Madrid. Spain; Servicio de Neurología. Hospital Ramón y Cajal de Madrid. Spain; Instituto Ramón y Cajal de Investigación Sanitaria (IRYCIS). Madrid. Spain; Servicio de Inmunología. Hospital Ramón y Cajal. Madrid. Spain; Instituto Ramón y Cajal de Investigación Sanitaria (IRYCIS). Madrid. Spain; Servicio de Neurología. Hospital Ramón y Cajal de Madrid. Spain; Instituto Ramón y Cajal de Investigación Sanitaria (IRYCIS). Madrid. Spain

## Abstract

Neurofilament light proteins (NFL) are a structural element of the neuronal cytoskeleton and are released with neuronal damage. Its levels are increased in cerebrospinal fluid (CSF) in the setting of neurodegenerative diseases. We investigated the CSF-NFL levels of Huntington´s disease (HD) patients (participating in a clinical trial SAT-HD) as well as of premanifest carriers and compared their results with a sample of healthy controls and correlated CSF-NFL levels with demographic and clinical variables (baseline demographic characteristics and HD measures of disease severity). CSF levels were significantly higher in all HD subjects [5014.4 (1557.3) ng/l] and pre-manifest carriers [1050 (212.13) ng/l as compared to controls [331.4 (200.2) ng/l] (p<0.00) and were correlated with age (correlation coefficient -0.37, p<0.01) and CAG triplet number (0,51, p<0.05) in the subset of HD patients. NFL levels were not correlated with age in the control group. We did not find any correlation with the remaining variables. These results indicate, as in previous studies, that CSF-NFL levels are a marker of neuronal damage in HD. It seems to be a highly sensitive, but non-specific marker of axonal damage. One of the limitations of our study is a very small number of patients in pre-symptomatic group and lack of individuals with very advanced HD. Further investigations should focus on study of CSF-NFL levels in advanced patients, tracking prospectively CSF-NFL levels and analysing its correlation with the clinical course and usefulness to monitor disease progression, validation and quantification of NFL levels in more accessible biofluids.

## Introduction

Neurofilament light proteins (NFL) are a structural element of the neuronal cytoskeleton and are released with neuronal damage. Its levels are increased in cerebrospinal fluid (CSF) in the setting of neurodegenerative diseases, including Alzheimer`s disease 1, frontotemporal dementia 2^, ^3 ,Huntington’s disease (HD) 4^, ^5 , and amyotrophic lateral sclerosis 6 , being considered a marker of neuronal damage. Recent studies have shown that NFL can also be reliably measured in blood and are a potential prognostic marker of neurodegeneration in patients with HD 7 , Alzheimer’s disease 8 . Moreover, NFL have been used as marker of neuronal injury in HIV- infection 9 , traumatic brain injury 10 and multiple sclerosis 11 . Also, NFL have revealed as a useful to monitor disease activity and response to treatment in multiple sclerosis^12^ .

HD is a paradigm of neurodegeneration in the need of appropriate biomarkers for monitoring disease progression. In previous studies various biomarkers of neuronal damage in blood and CSF were investigated, such as tau protein^13^, cytokines^14^, mitochondrial DNA^15^, mutant huntingtin protein^16^, low brain-derived neurotrophic factor (BDNF)^17^, different neurotransmitors^18^^, ^19^, ^20^, ^21, equilibrate nucleoside transporter ENT1^22^ as well as different markers of neuroinflammation^23^. Nevertheless, a number of studies were limited to animal models. To this time point, NFL seem to be the most promising and accessible biomarker of HD´s progression as the changes of level have been confirmed both in blood and CSF^4^^, ^7.

Two studies exploring NFL as biomarker in HD have been published in 2009 and 2017. Constantinescu et al. examined the levels of NFL in CSF of 35 HD patients in the setting of a clinical trial^4^. The CSF-NFL levels were significantly higher in HD subjects compared with age and gender matched controls, and were correlated with scores on the Unified Huntington’s Disease Rating Scale Total Functional Capacity (TFC), suggesting that NFL could be used as a potential biomarker. Recently, a study by Byrne et al. gave another, more practical perspective on this issue when levels of NFL were studied in blood^7^. They included subjects enrolled in TRACK-HD study, both pre-manifest and manifest carriers and matched healthy controls and correlated levels of NFL with different clinical parameters such as MRI neuroimaging findings, cognitive and motor evaluation and brain volume (global and regional). They were able to obtain blood sample at baseline and follow-up from 97 controls and 201 individuals with positive genetic testing and additionally compare it with NFL levels in CSF of 37 participants (23 mutation carriers and 14 healthy controls). All in all, they found that blood levels of NFL were higher in mutation carriers and correlated with disease staging as well as changes in MRI, cognitive decline and brain atrophy. What is even more interesting, elevated NFL in blood of pre-manifest carriers were associated with disease onset during the following 3 years. Concentrations of NFL in blood and CSF were correlated in mutation carriers. These results may result key in the setting of future trials.

In order to replicate previous published results we examined the CSF-NFL levels of HD patients (participating in a clinical trial SAT-HD), compared the results with a sample of healthy controls and correlated CSF-NFL levels with demographic and clinical variables (baseline demographic characteristics and HD measures of disease severity).

## Methods

The ELISA for NFL was performed according to previously published methods^24^. NFL-CSF levels were determined using commercial sandwich ELISA kits according to the manufacturers' recommendations (UmanDiagnostics AB, Umeå, Sweden). As specified in the trial protocol, a standardized lumbar puncture (LP) procedure was performed at the L4-L5 level in supine position in all cases. LP were performed at the end of period one and period two of the trial. Treatment assignment did not have impact on the NFL levels and no differences were found between CSF levels after period one and two. The samples analyzed correspond to the end of period one. Plasticware throughout the processing were of polypropylene material to minimize protein absorption. All the CSF samples were aliquoted and stored at -80º until assayed. In the HD group all the patients underwent the LP between 8:00 and 9:00am in a fasting state. The control samples were obtained from the Hospital Ramón y Cajal CSF Biobank collection. This samples were collected and stored according to the local ethical and legal requirements. All participants signed an informed consent form (ICF) for storing the samples at the Biobank for future research. All the controls met clinical criteria for “Symptomatic controls” (SCs) (patients with neurological symptoms, but have no objective clinical or paraclinical findings to define a specific neurological disease at the time of sampling). The time of the day, fasting status and collection vessels was not registered in any of the samples from the control group, however, all the samples were aliquoted and stored at -80º without delay after the LP. Also the storage time was longer (data not available) in the control group^25^.

Twenty-four EH participants (11 women; mean age 47.3 (SD 12.3)), two pre-manifest carriers and 34 controls (23 women; mean age 34.5 (SD 9.5)) were included. We compared levels of NFL-CSF levels with a regression model adjusted for age and gender. We examined the relationships (Pearson correlations) between NFL-CSF levels and demographic (age, gender) and clinical (CAG repeat number, disease burden, UHDRS motor, behavioural, functional total scores and NPI scale) variables. All the participants gave their signed consent for the procedure.

## Results

Demographic, clinical data and NFL-CSF levels of all participants are shown in Table 1. NFL-CSF results were significantly higher in all HD subjects [5014.4 (1557.3) ng/l] as compared to controls [331.4 (200.2) ng/l] (p<0.00) and were correlated with age (correlation coefficient -0.37, p<0.01) and CAG triplet number (0, 51, p<0.05) in the subset of HD patients. NFL levels were not correlated with age in the control group. We did not find any correlation with the remaining variables.

Figure 1. Demonstrates NFL-CSF concentration in different study subgroups, while Figure 2. shows correlations between NFL-CSF and age (Supplementary materials)

**Table 1: Demographic and clinical characteristics of participants.** 

group	n	male/female	age	CAG repeat length	Disease Burden	Total Functional Capacity	mUHDRS	mUHDRS
controls	34	11/23	34.5 (9.5)					331.4 (200.2)
Premanifest (early)	1	0/1	42	40	189.0	13	0	900
Premanifest (late)	1	1/0	45	41	247.5	13	0	1200
early	10	5/5	47.1 (12.9)	45.4 (2.6)	445.3 (102.3)	11.0 (0.6)	16 (6.7)	5416.0 (1734.4)
moderate	10	8/2	44.7 (12.8)	46.9 (4.6)	463.1 (125.4)	5.0 (1.0)	26 (9.1)	5416.0 (1734.4)
advanced	4	1/3	53.7 (8.8)	44.7 (2.9)	478.3 (93.3)	2.5 (0.5)	38 (10.2)	5416.0 (1734.4)

**Figure:**
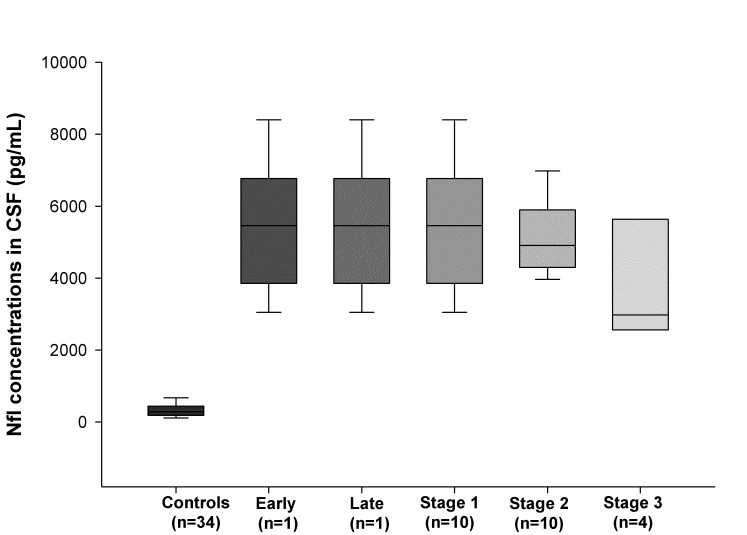
Fig 1. NFL-CSF concentration in different study subgroups

**Figure:**
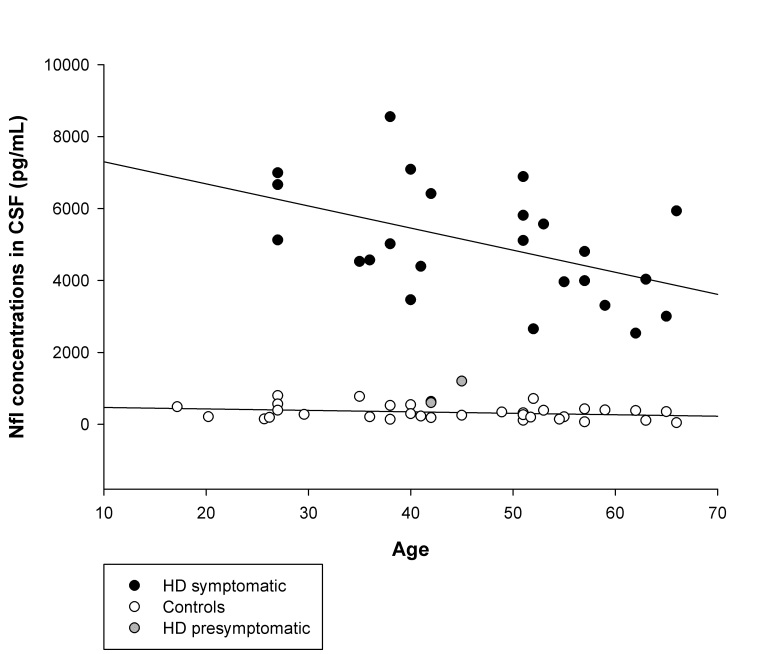
Fig 2. Correlations between NFL-CSF and age.

## Discussion

These results indicate, as in previous studies, that CSF-NFL levels are a marker of neuronal damage in HD. It seems to be a highly sensitive, but non-specific marker of axonal damage. One of the remaining questions is whether NFL shows superiority over other biomarkers. The biggest discussion considers total tau and NFL. Niemelä et al. published recently study aiming to compare those two biomarkers, but only in CSF^23^. They included in the analysis both 11 pre-manifest and 12 manifest carriers. While both biomarkers were correlated with each other and showed correlations with clinical parameters, NFL showed significant superiority over tau (TFC, r = -0.70 p < 0.01 for NFL vs r = -0.59 p < 0.01 for tau and Total Motor Score (TMS) r = 0.83p < 0.01 for NFL vs r = 0.67 p < 0.01 for tau). NFL was also significantly correlated with 5-year probability of disease onset, whereas tau was not. This strengths the thesis that rather NFL should be used in clinical trials although authors suggest that both could be used.

CSF-NFL levels were inversely correlated with age at CSF collection and directly correlated with CAG repeat number. We did not find any significant correlations between clinical assessment scores and NFL levels in HD subjects. Increased levels of NFL in younger patients may be an indicator of a more active neurodegenerative process in early stages of the disease. The transversal nature of our study is a limitation to our understanding of the progression of this biomarker.

All the HD patients included in the study were recruited for a clinical trial (SAT-HD with Sativex(®)) and only symptomatic, ambulatory HD patients were included. We were able to obtain samples from two more pre-manifest carriers, but this group was too small to carry out a valid comparative analysis. This limits our results to a subset of HD patients, excluding pre-symptomatic and advanced patients. Furthermore, control samples were collected in not homogenous conditions as we do not dispone with exact information about hour of collection and fasting status. That is why, there are differences in two study samples, the controls are younger than the HD population, and they had a narrower age range. This could be one an explanation why we did not find the widely replicated association between age and NFL.

Further investigations should be done in order to confirm preliminary results provided by us and previously published studies. In particular, CSF-NFL levels should be evaluated in pre-symptomatic, early symptomatic and advanced patients. Furthermore, CSF-NFL levels should be tracked prospectively and correlated with clinical tests, especially in perspective of usefulness to monitor disease progression. Although one study already confirmed practical application of NFL in blood, we are in strong need for validate quantification of NFL levels in accessible bio fluids such as blood, which would serve for longitudinal studies. Finally, it is necessary to evaluate NFL reactivity for therapeutic interventions and its potential to become an outcome for clinical trials in HD.

## Data availibility statement

The datasets generated during the current study are available in Dryad repository [www.datadryad.com, DOI: **doi:10.5061/dryad.j089n4j** ]. The data is associated to the title of the article.

## Authors roles

1. Research project: A. Conception: JLLS, B. Organization: JLS, C. Execution; JLLS, CP, JGC, JGY, JCAC

2. Statistical Analysis: A. Design: JLLS, B. Execution: JLLS, C. Review and Critique; JLLS

3. Manuscript Preparation: A. Writing of the first draft: JLLS, NSZ, B. Review and Critique; NSZ, JLLS

## Corresponding Author

**Correspondence to:**

José Luis López-Sendón Moreno

Servicio de Neurología. Planta 7D. Hospital Ramón y Cajal. 28034 Madrid. Spain.

Email: jlsendonmoreno@salud.madrid.org

Telephone number: 0034-913368821

## Conflict of interests

The authors have declared that no competing interests exist.

## Ethical Approval

The study was approved by the "Comité Etico de Investigación Clínica (CEIC) del Hospital Ramón y Cajal" (hospital's local Research Ethical Committe). SAT-HD trial was approved by the "Comité Etico de Investigación Clínica (CEIC) del Hospital Ramón y Cajal" (hospital's local Research Ethical Committe) and the Spanish Agency of Medicines. Comité Etico de Investigación Clínica (CEIC) del Hospital Ramón y Cajal" (Hospital's local Research Ethical Committe) and the Spanish Agency of Medicines approved the collection and storage of samples at the Hospital Ramón y Cajal CSF Biobank. All participants provided written informed consent for this study.

## References

[1] Rosengren LE, Karlsson JE, Karlsson JO, Persson LI, Wikkelsø C. Patients with amyotrophic lateral sclerosis and other neurodegenerative diseases have increased levels of neurofilament protein in CSF. J Neurochem. 1996 Nov;67(5):2013-8. PubMed PMID:8863508. 8863508 10.1046/j.1471-4159.1996.67052013.x

[2] Landqvist Waldö M, Frizell Santillo A, Passant U, Zetterberg H, Rosengren L, Nilsson C, Englund E. Cerebrospinal fluid neurofilament light chain protein levels in subtypes of frontotemporal dementia. BMC Neurol. 2013 May 29;13:54. PubMed PMID:23718879. 23718879 10.1186/1471-2377-13-54 PMC3671150

[3] Steinacker P, Semler E, Anderl-Straub S, Diehl-Schmid J, Schroeter ML, Uttner I, Foerstl H, Landwehrmeyer B, von Arnim CA, Kassubek J, Oeckl P, Huppertz HJ, Fassbender K, Fliessbach K, Prudlo J, Roßmeier C, Kornhuber J, Schneider A, Volk AE, Lauer M, Danek A, Ludolph AC, Otto M. Neurofilament as a blood marker for diagnosis and monitoring of primary progressive aphasias. Neurology. 2017 Mar 7;88(10):961-969. PubMed PMID:28179468. 28179468 10.1212/WNL.0000000000003688

[4] Constantinescu R, Romer M, Oakes D, Rosengren L, Kieburtz K. Levels of the light subunit of neurofilament triplet protein in cerebrospinal fluid in Huntington's disease. Parkinsonism Relat Disord. 2009 Mar;15(3):245-8. PubMed PMID:19056308. 19056308 10.1016/j.parkreldis.2008.05.012

[5] Wild EJ, Boggio R, Langbehn D, Robertson N, Haider S, Miller JR, Zetterberg H, Leavitt BR, Kuhn R, Tabrizi SJ, Macdonald D, Weiss A. Quantification of mutant huntingtin protein in cerebrospinal fluid from Huntington's disease patients. J Clin Invest. 2015 May;125(5):1979-86. PubMed PMID:25844897. 25844897 10.1172/JCI80743 PMC4463213

[6] Zetterberg H, Jacobsson J, Rosengren L, Blennow K, Andersen PM. Cerebrospinal fluid neurofilament light levels in amyotrophic lateral sclerosis: impact of SOD1 genotype. Eur J Neurol. 2007 Dec;14(12):1329-33. PubMed PMID:17903209. 17903209 10.1111/j.1468-1331.2007.01972.x

[7] Byrne LM, Rodrigues FB, Blennow K, Durr A, Leavitt BR, Roos RAC, Scahill RI, Tabrizi SJ, Zetterberg H, Langbehn D, Wild EJ. Neurofilament light protein in blood as a potential biomarker of neurodegeneration in Huntington's disease: a retrospective cohort analysis. Lancet Neurol. 2017 Aug;16(8):601-609. PubMed PMID:28601473. 28601473 10.1016/S1474-4422(17)30124-2 PMC5507767

[8] Fyfe I. Alzheimer disease: Neurofilament light in the blood marks Alzheimer degeneration. Nat Rev Neurol. 2017 May;13(5):257. PubMed PMID:28418024. 28418024 10.1038/nrneurol.2017.57

[9] Yilmaz A, Blennow K, Hagberg L, Nilsson S, Price RW, Schouten J, Spudich S, Underwood J, Zetterberg H, Gisslén M. Neurofilament light chain protein as a marker of neuronal injury: review of its use in HIV-1 infection and reference values for HIV-negative controls. Expert Rev Mol Diagn. 2017 Aug;17(8):761-770. PubMed PMID:28598205. 28598205 10.1080/14737159.2017.1341313

[10] Al Nimer F, Thelin E, Nyström H, Dring AM, Svenningsson A, Piehl F, Nelson DW, Bellander BM. Comparative Assessment of the Prognostic Value of Biomarkers in Traumatic Brain Injury Reveals an Independent Role for Serum Levels of Neurofilament Light. PLoS One. 2015;10(7):e0132177. PubMed PMID:26136237. 26136237 10.1371/journal.pone.0132177 PMC4489843

[11] Kuhle J, Disanto G, Lorscheider J, Stites T, Chen Y, Dahlke F, Francis G, Shrinivasan A, Radue EW, Giovannoni G, Kappos L. Fingolimod and CSF neurofilament light chain levels in relapsing-remitting multiple sclerosis. Neurology. 2015 Apr 21;84(16):1639-43. PubMed PMID:25809304. 25809304 10.1212/WNL.0000000000001491 PMC4409586

[12] Novakova L, Zetterberg H, Sundström P, Axelsson M, Khademi M, Gunnarsson M, Malmeström C, Svenningsson A, Olsson T, Piehl F, Blennow K, Lycke J. Monitoring disease activity in multiple sclerosis using serum neurofilament light protein. Neurology. 2017 Nov 28;89(22):2230-2237. PubMed PMID:29079686. 29079686 10.1212/WNL.0000000000004683 PMC5705244

[13] Rodrigues FB, Byrne L, McColgan P, Robertson N, Tabrizi SJ, Leavitt BR, Zetterberg H, Wild EJ. Cerebrospinal fluid total tau concentration predicts clinical phenotype in Huntington's disease. J Neurochem. 2016 Oct;139(1):22-5. PubMed PMID:27344050. 27344050 10.1111/jnc.13719 PMC5053298

[14] Bouwens JA, van Duijn E, Cobbaert CM, Roos RA, van der Mast RC, Giltay EJ. Plasma Cytokine Levels in Relation to Neuropsychiatric Symptoms and Cognitive Dysfunction in Huntington's disease. J Huntingtons Dis. 2016 Dec 15;5(4):369-377. PubMed PMID:27983562. 27983562 10.3233/JHD-160213

[15] Disatnik MH, Joshi AU, Saw NL, Shamloo M, Leavitt BR, Qi X, Mochly-Rosen D. Potential biomarkers to follow the progression and treatment response of Huntington's disease. J Exp Med. 2016 Nov 14;213(12):2655-2669. PubMed PMID:27821553. 27821553 10.1084/jem.20160776 PMC5110026

[16] Ciammola A, Sassone J, Cannella M, Calza S, Poletti B, Frati L, Squitieri F, Silani V. Low brain-derived neurotrophic factor (BDNF) levels in serum of Huntington's disease patients. Am J Med Genet B Neuropsychiatr Genet. 2007 Jun 5;144B(4):574-7. PubMed PMID:17427191. 17427191 10.1002/ajmg.b.30501

[17] Manyam NV, Hare TA, Katz L, Glaeser BS. Huntington's disease. Cerebrospinal fluid GABA levels in at-risk individuals. Arch Neurol. 1978 Nov;35(11):728-30. PubMed PMID:152621. 152621 10.1001/archneur.1978.00500350032006

[18] Manyam BV, Giacobini E, Colliver JA. Cerebrospinal fluid acetylcholinesterase and choline measurements in Huntington's disease. J Neurol. 1990 Aug;237(5):281-4. PubMed PMID:2146369. 2146369 10.1007/BF00314742

[19] Oepen G, Cramer H, Bernasconi R, Martin P. Huntington's disease - imbalance of free amino acids in the cerebrospinal fluid of patients and offspring at-risk. Arch Psychiatr Nervenkr (1970). 1982;231(2):131-40. PubMed PMID:6461312. 6461312 10.1007/BF00343834

[20] Klawans HL Jr. Cerebrospinal fluid homovanillic acid in Huntington's chorea. J Neurol Sci. 1971 Jul;13(3):277-9. PubMed PMID:4256172. 4256172 10.1016/0022-510x(71)90032-3

[21] Guitart X, Bonaventura J, Rea W, Orrú M, Cellai L, Dettori I, Pedata F, Brugarolas M, Cortés A, Casadó V, Chang CP, Narayanan M, Chern Y, Ferré S. Equilibrative nucleoside transporter ENT1 as a biomarker of Huntington disease. Neurobiol Dis. 2016 Dec;96:47-53. PubMed PMID:27567601. 27567601 10.1016/j.nbd.2016.08.013 PMC5102769

[22] Vinther-Jensen T, Börnsen L, Budtz-Jørgensen E, Ammitzbøll C, Larsen IU, Hjermind LE, Sellebjerg F, Nielsen JE. Selected CSF biomarkers indicate no evidence of early neuroinflammation in Huntington disease. Neurol Neuroimmunol Neuroinflamm. 2016 Dec;3(6):e287. PubMed PMID:27734023. 27734023 10.1212/NXI.0000000000000287 PMC5042104

[23] Niemelä V, Landtblom AM, Blennow K, Sundblom J. Tau or neurofilament light-Which is the more suitable biomarker for Huntington's disease? PLoS One. 2017;12(2):e0172762. PubMed PMID:28241046. 28241046 10.1371/journal.pone.0172762 PMC5328385

[24] López-Sendón Moreno JL, García Caldentey J, Trigo Cubillo P, Ruiz Romero C, García Ribas G, Alonso Arias MA, García de Yébenes MJ, Tolón RM, Galve-Roperh I, Sagredo O, Valdeolivas S, Resel E, Ortega-Gutierrez S, García-Bermejo ML, Fernández Ruiz J, Guzmán M, García de Yébenes Prous J. A double-blind, randomized, cross-over, placebo-controlled, pilot trial with Sativex in Huntington's disease. J Neurol. 2016 Jul;263(7):1390-400. PubMed PMID:27159993. 27159993 10.1007/s00415-016-8145-9

[25] Teunissen C, Menge T, Altintas A, Álvarez-Cermeño JC, Bertolotto A, Berven FS, Brundin L, Comabella M, Degn M, Deisenhammer F, Fazekas F, Franciotta D, Frederiksen JL, Galimberti D, Gnanapavan S, Hegen H, Hemmer B, Hintzen R, Hughes S, Iacobaeus E, Kroksveen AC, Kuhle J, Richert J, Tumani H, Villar LM, Drulovic J, Dujmovic I, Khalil M, Bartos A. Consensus definitions and application guidelines for control groups in cerebrospinal fluid biomarker studies in multiple sclerosis. Mult Scler. 2013 Nov;19(13):1802-9. PubMed PMID:23695446. 23695446 10.1177/1352458513488232

